# Differential interaction of Or proteins with the PSY enzymes in saffron

**DOI:** 10.1038/s41598-020-57480-2

**Published:** 2020-01-17

**Authors:** Oussama Ahrazem, Alberto José López, Javier Argandoña, Raquel Castillo, Ángela Rubio-Moraga, Lourdes Gómez-Gómez

**Affiliations:** 1 Instituto Botánico, Departamento de Ciencia y Tecnología Agroforestal y Genética, Universidad de Castilla-La Mancha, Campus Universitario s/n, 02071 Albacete, Spain; 2 VITAB Laboratorios, Polígono Industrial Garysol C/Pino, parcela 53, 02110 La Gineta, Albacete Spain

**Keywords:** Molecular biology, Plant sciences

## Abstract

Colored apocarotenoids accumulate at high concentrations in few plant species, where display a role in attraction of pollinators and seed dispersers. Among these apocarotenoids, crocins accumulate at high concentrations in the stigma of saffron and are responsible for the organoleptic and medicinal properties of this spice. Phytoene synthase and Orange protein are key for carotenoid biosynthesis and accumulation. We previously isolated four phytoene synthase genes from saffron with differential roles in carotenoid and apocarotenoid biosynthesis. However, the implications of Orange genes in the regulation of apocarotenoid accumulation are unknown. Here, we have identified two Orange genes from saffron, with different expression patterns. *CsOr-a* was mainly expressed in vegetative tissues and was induced by light and repressed by heat stress. Both *CsOr-a* and *CsOr-b* were expressed in stigmas but showed a different profile during the development of this tissue. The interactions of CsOr-a and CsOr-b were tested with all the four phytoene synthase proteins from saffron and with CsCCD2. None interactions were detected with CCD2 neither with the phytoene synthase 2, involved in apocarotenoid biosynthesis in saffron. The obtained results provide evidence of different mechanisms regulating the phytoene synthase enzymes in saffron by Orange for carotenoid and apocarotenoid accumulation in saffron.

## Introduction

In plants, carotenoids are C40 isoprenoid fat-soluble pigments synthesized in plastids. Carotenoids act as components of photosynthetic machinery, precursors for phytohormones (like abscisic acid (ABA) and strigolactones (SLs)), and are responsible for the yellow to reddish colors to many fruits and flowers^1^. The apocarotenoids are derived from carotenoids by the oxidative cleavage of specific double bond(s) over carotenoid precursors^2^. Some of these apocarotenoids are exploited by humans for their pigmentation properties, as the case of bixin^3^, heteranthin and ditaxin^4^, and saffron^5^. Saffron’s apocarotenoids are considered as bioactive components since they are able to treat chronic diseases and lowering risk of cancer and have positive effects on neurological disorders^6^.

The Or gene is present as a small gene family with at least two members, named Or-a and Or-b in different plant species^7^. This gene was discovered originally in cauliflower (*Brassica oleracea*; BoOr) where triggers the biogenesis of chromoplasts in non-green tissues^7^ without changing carotenoid biosynthetic gene expression^8^. This function of Or promoting chromoplast biogenesis for carotenoid accumulation, occur in multiple plant species^9–14^. The Or protein is target to plastids and contains a Cys-rich zinc finger domain, present in DnaJ co-chaperones, but lacking the J domain^15^. The Or proteins display holdase chaperone activity which protects and confers enhanced heat and oxidative stress tolerance^10^. In Arabidopsis, sweet potato, and melon, Or proteins have been shown to interact directly and regulate post-transcriptionally the phytoene synthase enzyme (PSY) and therefore controlling carotenoid biosynthesis^9,10,12^. In Arabidopsis, both Or proteins, Or-a and Or-b interact with PSY^12^.

PSY is the key regulatory enzyme in the biosynthesis of carotenoid where catalyzes the first committed reaction to produce phytoene. In the case of Arabidopsis and other Brassicaceae only one *PSY* gene (Arabidopsis genome Initiative, 2000) is found in the genome, while two or more *PSY* genes have been reported in other plants, including the grasses and other crops of economic importance^16,17^. The presence of different PSY enzymes in some of these plants is associated to a specific specialization for the high production of carotenoids in chromoplast-containing tissues, to stress responses, or with the establishment of mycorrhization^17^. However, data on the interaction of the two distinct Or proteins with the different PSY enzymes are missing from studies performed in these species such tomato^11^ or sweet potato^10^.

*Crocus sativus* accumulates exceptional high levels of polar apocarotenoids known as crocins, which confer the color to the saffron spice^5^. The biosynthesis and accumulation of crocins take place in the stigma and is developmentally regulated. Young undeveloped stigma in saffron and in other Crocus species is white, containing amyloplasts^18,19^. The transition from amyloplast to chromoplast marks the initiation of crocins biosynthesis and their accumulation in the stigma^18^ with the presence of a distinctive red color. Crocins biosynthesis in saffron involves a chromoplast-specific carotenoid biosynthetic pathway^20–22^, where CsPSY2 is the enzyme catalyzing the first committed step in the carotenoid biosynthesis for the production of the colored apocarotenoids that accumulate in the stigma of saffron, together with CsLCY-B2, CsBCH2 and CCD2 enzymes^20,21,23^. Thus, the expression of all the four genes encoding for these four enzymes contributing to the apocarotenoid biosynthesis in the saffron stigma, coordinately increases during its development^17,20,21,24^, resulting in a strong activation of the metabolic flux to the apocarotenoid pathway. In addition to *CsPSY2*, three additional *PSY* genes are present in saffron^22^ displaying specialized functions in carotenoid and apocarotenoid homeostasis. *CsPSY1a* and *CsPSY1b* are mainly expressed in photosynthetic tissues, but are also involved in stress responses, while *CsPSY3* is related to strigolactones production^17^.

In this study, we characterized the Or proteins from saffron, and studied their expression in different tissues at different developmental stages, and under different stress conditions. In addition, we analyzed the interaction of these two Or proteins with the four different CsPSY enzymes. CsOr-b was not interacting with any of the enzymes tested, whereas CsOr-a showed differential interactions. Weak interaction was detected between the CsOr-a and CsPSY2, but nor with CsCCD2, suggesting that Or is not a factor influencing on apocarotenoid biosynthesis in the stigma of saffron.

## Results

### Properties of saffron Or proteins

**Figure 1:**
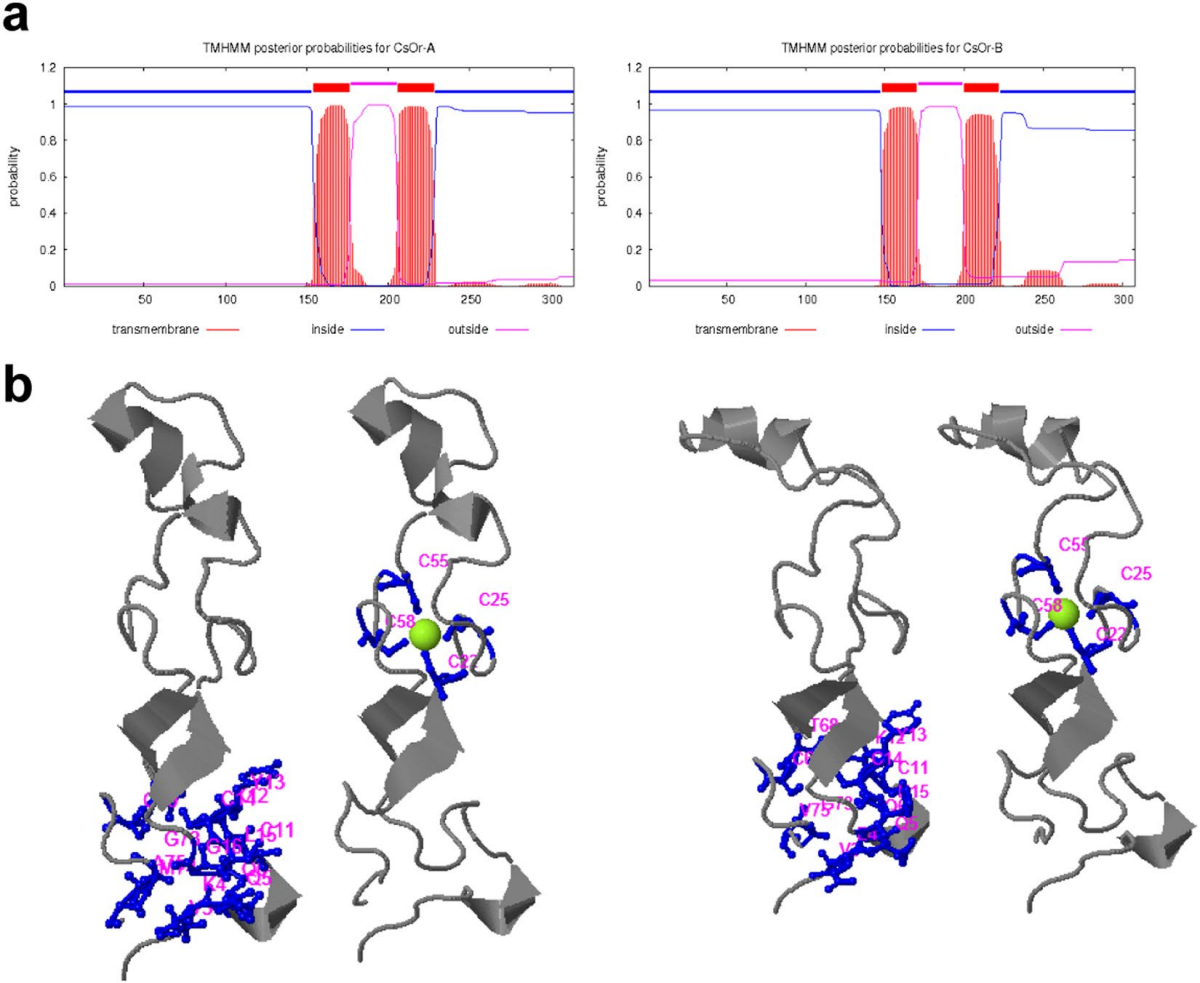
Structural details in the OR sequences from saffron. (**a**) Putative hydrophobic profiles of CsOr-a and CsOrb, evaluated by the TMHMM Server v. 2.0. (**b**) Tridimensional models of the CsOr enzymes with their co-factor and the amino acids residues involved in the interaction in blue obtained with COACH (https://zhanglab.ccmb.med.umich.edu/COACH/).

The *CsOr-a* and *CsOr-b* genes were initially identified from transcriptomes of saffron^24–26^. Both genes encode proteins of 314 and 308 amino acids, respectively. As shown in Suppl. Fig. 1, the overall amino acid identity of the two Or is 66.67%. The C-terminal region of these two Or proteins (starting at amino acid 70 in the CsOr-a and CsOr-b amino acid sequences) are highly conserved, with 76.13% identity. The large divergence in the N-termini is partially due to the plastid transit peptides. ChloroP predicted transit peptides of 39 and 49 amino acids for CsOr-a and CsOr-b, respectively. Both proteins showed the presence of two transmembrane domains and a Cys-rich zinc finger domain (Fig. 1a,b).

**Figure 2:**
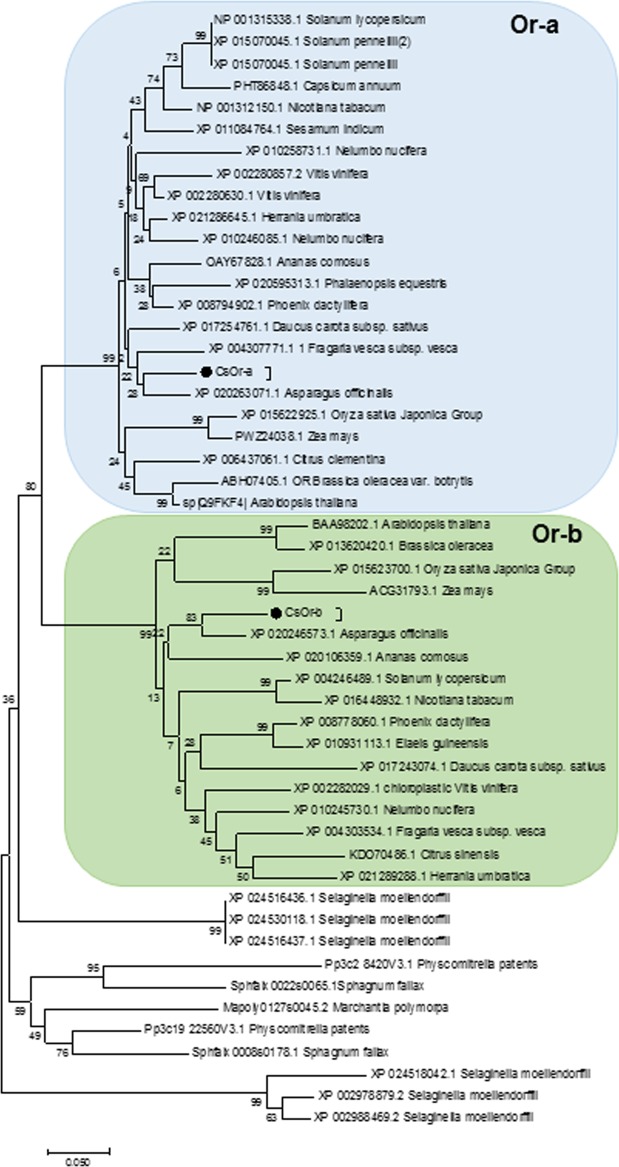
Phylogenetic analyses Or sequences identified in saffron. Dendogram of CsOr-a and CsOr-b amino acid sequence with Or proteins from different plants species. The blue and green background contain the clusters including Or-a and Or-b sequences.

A comparison of both saffron Or proteins with Or orthologs revealed that they are in separate clusters (Fig. 2), as shown for the Or present in other plant species. We generated LOGOs for these two sequences classes, and observed the conservation of key residues as the presence of the DnaJ-like cysteine-rich zinc finger domain (Suppl. Fig. S2), suggesting that they may have originated from one gene in an ancestral species. Inside the Or-a and Or-b clusters, it can be observed that the proteins did not cluster in independent groups depending on whether the plants are monocotyledonous or dicotyledonous (Fig. 2), indicating that the main characteristics of *Or-a* and *Or-b* in plants were established before the dicot-monocot split. In addition, we also determine intron number and mRNA variants of *Or* genes in different plant species (Tables 1 and 2). Intron number in *Or-a* genes was between 7–8, and their positions were highly conserved, while in *Or-b* the genes mainly showed 7 introns and their positions were also conserved (Tables 1 and 2; and Suppl. Figs. 3 and 4). These results also suggest that the basic exon/intron structure of both subfamilies developed before the monocot and dicot split, although some losses might have occurred.

**Table 1:** Number of intronic sequences present in Or-a genes from different plant species.

Or-a genes accession number	Plant species	intron	variants
LOC107776698	*Nicotiana tabacum*	8	1
LOC107819685	*Nicotiana tabacum*	7	2
LOC101244466	*Solanum lycopersicum*	7	1
LOC107863565	*Capsicum annuum*	7	1
LOC105166943	*Sesamum indicum*	7	1
LOC113727348	*Coffea arabica*	7	1
LOC113725223	*Coffea arabica*	7	1
LOC114293474	*Camellia sinensis*	8	1
LOC100266582	*Vitis vinifera*	7	1
LOC100261377	*Vitis vinifera*	8	2
LOC18103854	*Populus trichocarpa*	7	1
LOC115705453	*Cannabis sativa*	7	1
LOC109839048	*Asparagus officinalis*	8	2
LOC111397279	*Olea europaea*	8	2
LOC104598393	*Nelumbo nucifera*	8	1
LOC104589446	*Nelumbo nucifera*	7	1
LOC110599866	*Manihot sculenta*	7	2
LOC110617496	*Manihot sculenta*	7	1
LOC18044280	*Citrus clementina*	7	1
LOC110905010	*Helianthus annuus*	7	2
LOC103710788	*Phoenix dactilyfera*	8	2
LOC108224601	*Daucus carota*	7	1
LOC4330168	*Oryza sativa*	7	1
LOC100275801	*Zea mays*	7	1
LOC8077677	*Sorghum bicolor*	7	1
LOC111892120	*Lactuca sativa*	7	1
LOC112520643	*Cynara cardunculus*	8	1
LOC104906344	*Beta vulgaris*	7	1
At5g61670	*Arabidospis thaliana*	7	1

**Table 2:** Number of intronic sequences present in Or-b genes from different plant species.

Or-b gene accession number	Plant species	intron	variants
LOC111399623	*Olea europaea*	7	1
LOC100261394	*Vitis vinifera*	7	1
LOC111882594	*Lactuca sativa*	7	1
LOC105161741	*Sesamum indicum*	7	1
LOC113308095	*Papaver somniferum*	7	1
LOC112518601	*Cynara cardunculus*	7	1
LOC104589196	*Nelumbo nucifera*	7	1
LOC110873310	*Helianthus annuus*	7	1
LOC10488328	*Beta vulgaris*	7	1
LOC113697311	*Coffea arabica*	8	4
LOC110729745	*Chanopodium quinoa*	7	2
LOC110730979	*Chanopodium quinoa*	7	1
LOC114318605	*Camellia sinensis*	7	2
LOC109824395	*Asparagus officinalis*	7	2
LOC111882594	*Lactuca sativa*	7	1
LOC1000261394	*Vitis vinifera*	7	1
LOC111299623	*Olea europaea*	7	1
AT5G06130.2	*Arabidospis thaliana*	6	1
LOC100275820	*Zea mays*	7	1
LOC4329565	*Oryza sativa*	7	1
LOC8064223	*Sorghum bicolor*	7	1
LOC7491929	*Populus trichocarpa*	7	1
LOC115701039	*Cannabis sativa*	7	1
LOC18039750	*Citrus clementina*	7	1
LOC108215199	*Daucus carota*	7	1
LOC103697897	*Phoenix dactylifera*	7	1
LOC101247247	*Solanum lycopersicum*	7	1
LOC107774004	*Nicotiana tabacum*	7	1

### Tissue specificity of saffron Ors

**Figure 3:**
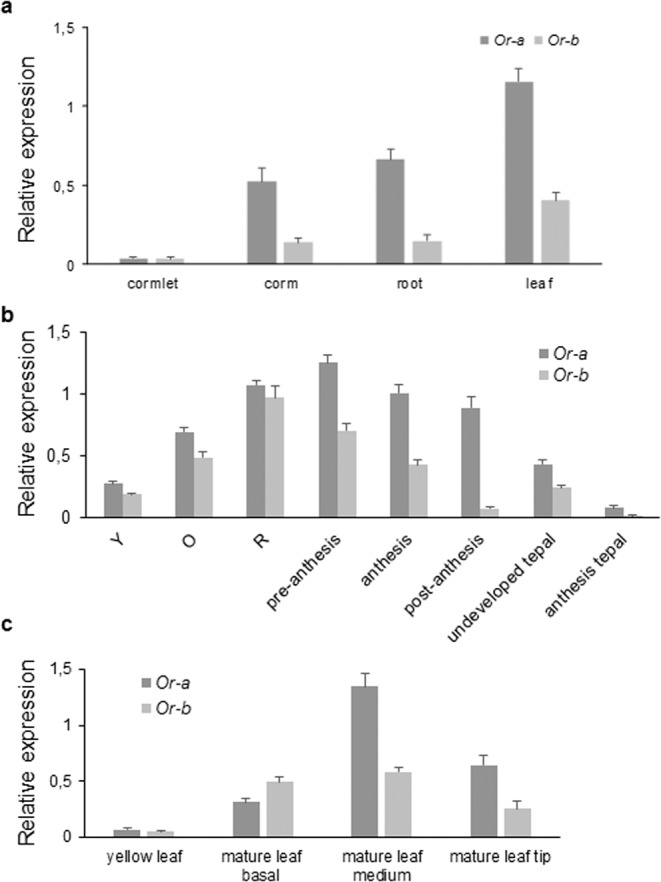
Relative expression levels of the *CsOr* genes in vegetative and reproductive tissues investigated by qRT-PCR. (**a**) Transcripts levels in corms at two different developmental stages, and in the root and developed leaves. (**b**) Expression levels in six developmental stages of the stigma and in undeveloped and fully developed tepals. (**c**) Transcripts levels in leaves at different developmental stages and along the mature leaf. Scale bars indicate five biological replicates ± SD.

Real-time qRT-PCR was used to investigate the expression levels of both *CsOr* genes in different tissues (leaves, roots, and corm) and in developing and mature stigmas, tepals and fully developed leaves. Transcripts levels of *CsOr-a* were higher than those of *CsOr-b* in all the tested tissues, although the opposite was found on certain developmental stages. The highest expression levels of *CsOr-a* and *CsOr-b* were found in leaves (Fig. 3a), followed by stigmas (Fig. 3b) in the preanthesis and in the red stages for *CsOr-a* and *CsOr-b*, respectively (Fig. 3b). Expression levels in tepals, were higher for both genes in white undeveloped teal than in lilac tepals at anthesis (Fig. 3b). The expression levels in leaves were investigated in more detail by the dissection of leaves along their long axis (Fig. 3c). In the basal part of the mature leaf, characterized mainly by the presence of proplastids, *CsOr-b* expression was higher than in *CsOr-a* (Fig. 3c). The transcript levels of *CsOr-a* are highly increased in the medium part of the leaf (Fig. 3c), where the formation of plastid structures is taking place. However, at the leaf tip, characterized by the presence of mature photosynthetic cells and fully developed chloroplast, the expression levels of both genes decreased (Fig. 3b), but their expression levels were not as low as the ones observed in pale undeveloped yellow leaves (Fig. 3b).

### Stress regulation of saffron Or proteins

**Figure 4:**
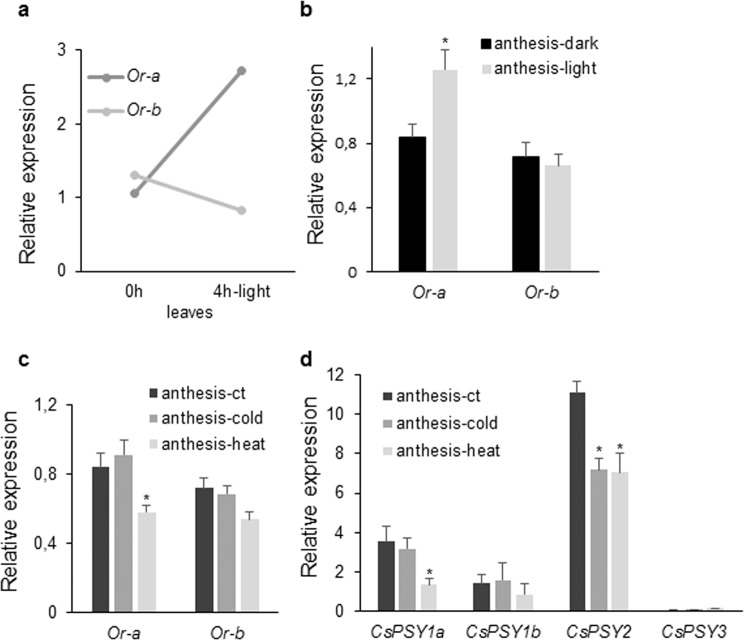
Relative expression levels of the *CsOr* genes under different stress conditions investigated by qRT-PCR. (**a**) Transcript levels of *CsOr* genes in leaves dark-adapted for 2 h and upon illumination with white light for 4 h. (**b**) Transcript levels of *CsOr* genes in red stigmas in dark and light conditions during 24 h. (**c**) Expression levels of *CsOr* genes in stigmas subjected to cold and heat treatments during 24 h. (**d**) Expression levels of *CsPSY* genes in stigmas subjected to cold and heat treatments during 24 h. Scale bars indicate five biological replicates ± SD. Asterisks mark statistically significant differences (p < 0.05) relative to controls.

Further, the effect of light on *CsOr-a* and *CsOr-b* expression was tested in leaves and stigmas (Fig. 4a,b). In both tissues only *CsOr-a* expression was clearly upregulated by light (Fig. 4a,b), while *CsOr-b* expression was not affected.

Cold and heat treatments were also performed on stigmas at anthesis in order to determine the effects of these treatments on the expression levels of *CsOr* and *CsPSY* genes (Fig. 4c,d). Heat stress induced a reduction in the expression levels of *CsOr-a* and *CsOr-b*, and this reduction was more evident for *CsOr-a* (Fig. 4c). Cold and heat treatments have a negative effect by downregulating the expression of *CsPSY2* in stigmas at anthesis. However, the levels of expression of *CsPSY1a* and *CsPSY1b* were only affected by the heat treatment (Fig. 4d).

### Interaction of CsOr proteins with CsPSY

**Figure 5:**
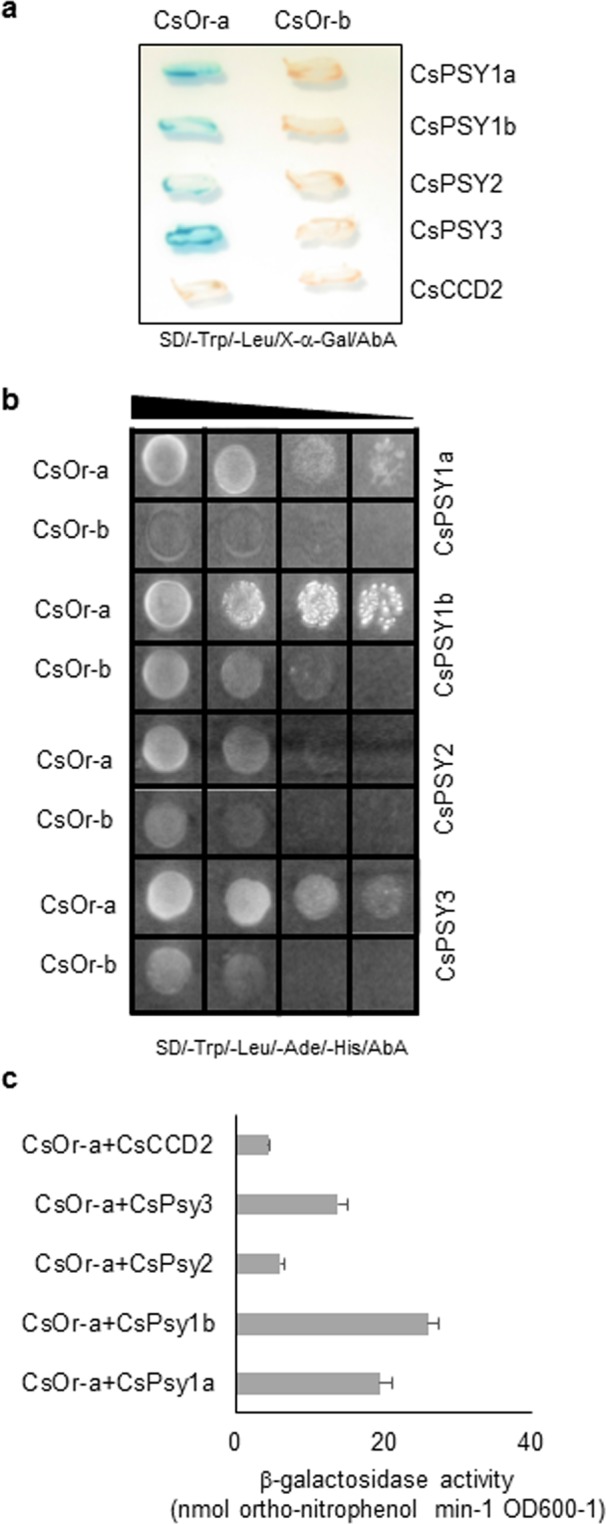
CsOr-a interacts with CsPSY enzymes. Yeast two-hybrid assays for CsOr-a and CsOr-b interaction with CsPSYs and CsCCD2. CsOr-a or CsOr-b was fused to the activating domain (AD), and CsPSY1a, CsPSY1b CsPSY2, CsPSY3 or CsCCD2 was fused to the binding domain (BD). (**a**) Yeast cells transformed with different combinations of constructs were grown on a chromogenic selective medium (SD/-Trp/-Leu/X-α-Gal/AbA). (**b**) Yeast cells transformed with different combinations of constructs of Or and PSY proteins were spotted on selective medium (SD/-Ade/-His/-Leu/-Trp), supplemented with Aureobasidin A (AbA). (**c**) β-galactosidase assays of in *vivo* interactions between CsOr-a and CsPSY and CsCCD2 proteins. Means and se from three biological replicates are shown.

To determine whether CsOr-a and CsOr-b interact or not with each of the different CsPSY enzymes, we performed yeast two-hybrid analysis combining each Or protein with the four CsPSYs and CsCCD2. The CsPSY1a, CsPSY1b, CsPSY2 and CsPSY3 were fused to Gal4 DNA-BD, while CsOr-a and CsOr-b were fused to Gal4-AD. On selective medium (SD/-Trp/-Leu/X-α-Gal/AbA), only diploid cells expressing both PSY and Or show growth and those which have a direct interaction display blue coloration (Fig. 5a). In all cases, blue coloration was detected for CsOr-a and all the tested proteins except for CsCDD2 (Fig. 5a), while the CsOr b did not show coloration with the analyzed genes.

We plated the obtained diploid yeast cells in the higher stringency selective medium (SD/-Trp/-Leu/-Ade/-His/AbA) to corroborate the interactions previously observed. A stronger interaction was observed between CsPSY1a + CsOr-a, CsPSY1b + CsOr-a and CsPSY3 + CsOr-a, (Fig. 5b), and weak interaction was detected between CsPSY2 and CsOr-a (Fig. 5b). Quantitative β-galactosidase activity assays showed that CsPSY1a and CsPSY1b had the strongest interaction with CsOr-a among the four CsPSYs (Fig. 5c).

## Discussion

The natural insertion of a copia-like retrotransposon in the cauliflower BoOr gene induced carotenoid accumulation in non-colored plant tissues^7^. After this discover, a new SNP *Or* mutant was reported to drive β-carotene accumulation in melon fruit flesh^27^. Both mutations induced the formation of chromoplasts containing carotenoid sequestering structures^7,11,28^. These mutated versions of Or have been shown to boost carotenoid accumulation in different plant species^11,29–31^. However, the Or gene mechanism of action in the regulation of carotenoid accumulation is not clear yet^9,12^. The Or protein displayed holdase chaperone activity and enhances PSY protein stability^10,12^, counterbalancing Clp-mediated proteolysis of PSY in plastids^32,33^. However, this regulation of PSY levels and activity was not the mechanism by which an Or natural allelic variation in melon governs fruit β-carotene levels. In this case, the Or mutation stabilizes β-carotene and inhibits its turnover, resulting in the accumulation of β-carotene in the chromoplast^9^. These data indicate several roles of Or in carotenoid accumulation: acting as a posttranscriptional regulator of PSY, in the biogenesis of chromoplasts, and inhibiting carotenoid metabolism downstream of β-carotene.

The *Or* gene family is represented by at least two members of two different classes (named as *Or-a* and *Or-b*) in all the different plant species analyzed. The duplication that yielded these two genes occurred before the divergence of monocots and dicots. The two genes encoding for Or in *Crocus* were detected in all the tissues analyzed, with higher levels of expression in mature leaves and stigma. However, differences in both tissues were observed depending on the developmental stage. *CsOr-b* expression was higher in the stigma tissue than in leaves, and reached the highest levels of expression in the red stage of the stigma, from this stage in advance the expression levels dropped. In leaves, *CsOr-b* was expressed at higher levels in the mid-part of the mature leaf, but the expression levels were lower than in the stigma. By contrast, *CsOr-a* was expressed at similar levels in the stigma in the pre-anthesis stage and in the mid-part of mature leaves. The function of Or has been associated to the differentiation of proplastids and/or other noncolored plastids into chromoplasts, providing a metabolic sink for carotenoid accumulation^7^. During the development of the stigma in saffron, the increase in size is concomitant with the transformation of amyloplasts into chromoplasts^18^. During the transition from the red to the pre-anthesis stage the amyloplast became undetectable^18^, and the number of plastoglobules and a membranous network increased^34^, matching the fully developed chromoplasts. This process is associated with the expression levels observed for *CsOr-a* and *CsOr-b*. The expression levels of both genes were similar to the ones previously observed for *CsPSY1a* and *CsPSY1b* in stigmas^17^. However, while all the PSY enzymes from saffron, with the exception of CsPSY3, have been detected in a chromoplast proteome obtained from red stigmas of saffron, no peptides for Or were identified^18^, which could be most probably due to its topological location within the chromoplast or to a low protein abundance. The same has been observed in the chromoplast of other plant species, where Or was undetectable in water melon, tomato, cauliflower or papaya^35,36^. The mismatch between protein and transcript abundance point out to a posttranscriptional regulation of Or in these tissues.

In the case of leaves, chloroplast development from the proplastid to functional chloroplasts is observed as a gradient along the leaf blade, with fully developed chloroplast at the tip of the leaf^37^, and the presence of etio-chloroplast and amyloplast in the yellow part of the leaf^34^. The highest expression levels of *CsOr-a* and *CsOr-b* were associated with the development of chloroplast, supporting the involvement of Or in the development of the chloroplast structure in leaves^7^. Further, *CsOr-a* expression was associated to light stress, while *CsOr-b* was unresponsiveness. Similarly, *CsPSY1b* was as well linked to photoprotection, while *CsPSY1a* was insensitive^17^. Interestingly, the strongest interaction was observed between CsOr-a and CsPSY1b.

Expression levels of some genes encoding for plant DnaJ which are targeted to the chloroplast are induced by different abiotic stresses^38–40^. In addition, in sweet potato *Or* expression was differentially affected by salt, drought, oxidative stress and heat stress, depending on the analyzed tissue^10,41–43^. In sweet potato, *Or* was downregulated by heat stress in leaves, as the case of *CsOr-a* and *CsOr-b* in heat-treated stigmas. Similarly, heat stress downregulated *CsPSY1a* and *CsPSY1b* expression levels. *CsPSY2* expression in stigmas was downregulated by heat and cold, while *CsCCD2* was downregulated by heat but upregulated by cold^24^.

The two Or proteins from Arabidopsis are functionally redundant and both able to interact with the Arabidopsis PSY enzyme^12^, and such interaction has been also proved in sweet melon between the PSY1 enzyme and Or-a^10^. Many other proteins have been identified as putative interacting proteins with Or, among them components of the photosynthesis machinery, transcription factors and other chaperones^12,44^. We observed strong interactions for CsOr-a with CsPSY1a, CsPSY1b and CsPSY3, and a weak interaction with CsPSY2. Interestingly, when various physicochemical properties of the CsPSY enzymes were computed, similar amino acid compositions were observed (Suppl. Fig. 5). However, major differences were observed at the level of protein stability. CsPSY2 with an instability index smaller than 40 was the only CsPSY predicted as stable (Suppl. Fig. 5). This fact could explain the weak interaction of CsPSY2 with CsOr-a, since Or functions as a chaperone, stabilizing PSY to greatly reduce PSY protein turnover rate^10,12,32^, the strong interactions with CsPSY1a, CsPSY1b and CsPSY3 may be explained by an increase of the stability of these proteins, however, CsPSY2 is stable by itself.

By contrast, no interactions were observed between CsOr-b and the CsPSY enzymes. CsOr-a and CsOr-b showed 66.67% identity, mainly due to differences in the N-terminal region, and after the removal of the predicted transit peptides the identity increased up to 72% (Suppl. Fig. 6). In sweet potato, protein deletion studies showed that IbPSY interact with the IbOr-N fragment (1-232 amino acids), which contains the N-terminal unknown region (30–153 amino acids) and the transmembrane domains (154–232 amino acids). The transmembrane domain showed high degree of conservation among the Or proteins (Suppl. Fig. 6). Major differences between CsOr-a and CsOr-b are observed in the N-terminal unknown region, suggesting that this N-terminal region could determine the interaction with the PSY proteins, in fact in melon, the N-terminal 162 amino acids are sufficient to produce an interaction with PSY^9^. Closer analyses of the N-terminal regions of the Or proteins from Arabidopsis and Crocus (Suppl. Fig. 7), showed lower content in hydrophobic amino acids in CsOr-b. This reduction could explain the absence of interaction observed for CsOr-b with the PSY proteins of saffron.

In sweet potato, the Or protein also specifically interacts with the carotenoid cleavage dioxygenase CCD4^45^, suggesting an important role in maintaining carotenoid homeostasis, perhaps by negatively regulating the CCD4 activity and allowing carotenoids accumulation^46^. The possible interaction of CsCCD2 with CsOr-a and CsOr-b was tested, however, in both cases none of the CsOrs were able to interact, suggesting that CsOrs in saffron are not involved in controlling apocarotenoid biosynthesis and accumulation, which can explain as well the weak interaction of these proteins with CsPSY2. Further, the Or gene in *C sieberi* is not differentially expressed in apocarotenoid-containing tissue sectors^19^, providing additional data on the implications of Or on apocarotenoid deposition in Crocus species.

## Methods

### Plant material

*Crocus sativus* plants were used in this study. *C. sativus* was grown outdoors in the Botanical Garden of Castilla-La Mancha (JBCM). Light and other stress treatment were performed as previously described^17,24,47^.

### Nucleic acid purification and cDNA isolation

Total RNA was isolated from red stigmas^48^, following the manufacturer’s protocols (Qiagen, Hilden, Germany). First-strand cDNAs were synthesized by RT from 2 µg of total RNA using an 18-base pair oligo dT primer and a first-strand cDNA synthesis kit (GE Healthcare Life Sciences, www.gelifesciences.com) according to manufacturer’s instructions. These cDNAs were used as templates for PCR using specific primers for *CsOr-a* and *CsOr-b* (Suppl. Table 1). Thermal cycling parameters were 2 min at 95 °C, 35 cycles of 20 s at 95 °C, 20 s at 60 °C and 1 min 30 s at 72 °C, followed by a final extension of 5 min at 72 °C. The PCR products were separated in a 1% agarose gel, purified, ligated into pGEM-T (Promega, www.promega.com), and then introduced into *E. coli*.

### DNA sequencing and analysis of DNA and protein sequences

Sequencing was done by using an automated DNA sequencer (ABI PRISM 3730xl, Perkin Elmer, Macrogen Spain, www.macrogen.com), as previously described^49^. Similarity searches were done with BLAST (NCBI; http://www.ncbi.nlm.nih.gov), motif searches with PROSITE (http://expasy.hcuge.ch/sprot/prosite.html), SignalP (http://www.cbs.dtu.dk/services/SignalP), and TMPRED (http://www.isrec.isb-sib.ch/sofware/sofware.html), structural analyses with ProtParam (https://web.expasy.org/protparam/). Logos were generated using the Seq. 2Logo server (http://www.cbs.dtu.dk/biotools/Blast2logo-1.1/). Phyre server modelling (http://www.sbg.bio.ic.ac.uk/phyre2/) was done using chaperone Chain: A: PDB Molecule:dnaj/hsp40 cysteine-rich domain superfamily protein crystal structure of the bsd2 homolog of *Arabidopsis thaliana*, which showed confidence 99.0% and coverage:19%.

### Phylogenetic analysis

The amino acid sequences were aligned using the BLOSUM62 matrix with the CLUSTALW (http://www.clustal.org) algorithm-based AlignX module from MEGA Version 6.0 (http://www.megasoftware.net/mega.html), and used to generate a neighbour-joining tree with bootstrap support (5000 replicates). Gaps were deleted pairwise.

Comparative analysis of gene structures was done with the comparative genomics database PIECE (http://wheat.pw.usda.gov/piece) for Plant Intron and Exon Comparison^50^.

### RNA extraction and quantitative RT-PCR analysis

Total RNA was extracted with the RNeasy Plant Mini Kit following the manufacturer’s instructions (Qiagen, Hilden, Germany). First-strand cDNAs were synthesized as previously described^51^. The cDNAs samples were used as templates in RT-qPCR assays in the presence of a SYBR Green PCR master mix in an Applied Biosystems 7900HT Fast Real-Time PCR system (Applied Biosystems, Foster City, CA) (Suppl. Table 1). Thermal cycling conditions consisted of a first step of denaturation at 95 °C during 5 min, followed by 40 cycles at 94 °C for 20 s, 58 °C for 20 s, 72 °C for 20 s, and a final extension at 72 °C for 5 min. A StepOne™ Thermal Cycler (Applied Biosystems, Foster City, California, USA) was used and results analyzed with StepOne software v2.0 (Applied Biosystems, Foster City, California, USA). DNA melt curves were developed for each primer combination to confirm the presence of a unique product. Transcript levels of the CsOr genes were normalized with those of RPS18^47^. Relative expression levels were calculated using the 2-ΔΔCt method^52^. Analysis of all gene expression was run in triplicate with three biological repeats. Statistical analyses were done as previously described^53^.

### Two-hybrid assay

The ORFs were truncated by the putative transit peptides as predicted by chloroP (ref). cDNA sequences of *CsPSY1a*, *CsPSY1b*, *CsPSY2*, *CsPSY3*, *CsOr-a*, and *CsOr-b* without the sequences encoding their transit peptides were cloned into pGBKT7 or pGADT7 to make either BD or AD fusions (see Table S1 for primer information). Plasmids were transformed into yeast strain Y2HGold (BD) or Y187 (AD) and mated with each other following manufacturer instructions (Clontech, Takara Bio USA). Cells carrying BD fusions were mated with strains carrying AD fusions. The resulting diploid cells were grown and subcultured in synthetic complete medium lacking leucine and tryptophan (SD/-Leu/-Trp) and supplemented with Aureobasidin A and X-α-Gal. Genuine interaction tests were performed with yeast cells spotted on selective medium (SD/-Ade /-His/-Leu/-Trp), supplemented with Aureobasidin A in a series of 1:10 dilutions starting with an OD_600_ = 2 after two days growth at 29 °C.

For β-galactosidase activity assays, yeast cells were cultivated overnight in YPD. Culture of 500 μL was pelleted, frozen in liquid nitrogen and resuspended in 1 mL buffer H (100 mM HEPES/KOH, pH 7.0, 150 mM NaCl, 2 mM MgCl_2_, and 1% BSA). Cells were disrupted with chloroform and SDS. A total of 200 μL of ortho-nitrophenyl-β-galactoside in buffer H at 4 mg/mL was added and incubated at 30 °C until the mixture turned yellow. The reaction was stopped by adding 500 μL 1 M Na_2_CO_3_ and concentration of ortho-nitrophenol in the supernatant was determined photometrically at 420 nm using the molar extinction coefficient of ortho-nitrophenol = 3300 g l^−1^ Mol^−1^. β-Galactosidase activity was calculated as nmol ortho-nitrophenol min^−1^ OD_600_^−1^. The β-galactosidase assays were done in triplicate.

## Electronic supplementary material

Supplementary information.
