# Supplementary material for: Differential interaction of Or proteins with the PSY enzymes in saffron
Source: Sci Rep. 2020 Jan 17;10:552. doi: 10.1038/s41598-020-57480-2 (PMC6969158; doi:10.1038/s41598-020-57480-2)
Supplement: Supplementary file 1 — Supplementary information. [file 41598_2020_57480_MOESM1_ESM.pdf]

Differential interaction of Or proteins with the PSY enzymes in saffron

Oussama Ahrazem[1], Alberto José López[1], Javier Argandoña[1], Raquel Castillo[2], Ángela Rubio-Moraga[1], Lourdes Gómez-Gómez[1,*]

Supplemental Figure 1

```
• CsOr-a      MLFSSRILTCSYSPIPLNSSRWSRPPD--------RGIIWKQRWRSMASADPDASSSFAQ     52
• CsOr-b      M-----ASISSFSLLPPPNSRFSLPAPSKTHVLFSDGKSWSSHRRIPAGIRPFRCSS---     52
•             *        .*:* :*  .**:* *           *  *..: *  *.  *  .**
•
• CsOr-a      SVDSPATGETTDKNQPGFCIIEGPETVQDFAKMELQEMQDNIRSRRNKIFLHMEEVRRLR     112
• CsOr-b      ----GEDTIPGGDNGASFCIIEGPETVQDFVQMQFQEIQDNIRSRRNKIFLLMEEVRRLR     108
•                        ..*  .*************.:*::**:************* ********
•
• CsOr-a      IQQRIKNAELGIVKEEQESELPDFPSFIPFLPPLTPANLKQYYATCFSMIAGIIVFGGLL     172
• CsOr-b      VQQRIKSSE--IIDESEENEMPDMRSTIPFLPSVTPKTLKQLYLTSFSFVSGIIVFGGLL     166
•             :*****.:*  *:.*.:*.*:**: * ***** :** .*** * *.**:::*********
•
• CsOr-a      APALELKLGIGGTSYEDFIRNMHLPMQLSQVDPIVASFSGGAVGVISALMVVEINNVKQQ     232
• CsOr-b      APVLELKLGLGGTSYEDFIRSVHLPLQLSQVDPIVASFSGGAVGVISALMLVEANNVEQQ     226
•             **.******:**********.:***:************************:** ***:**
•
• CsOr-a      EHKRCKYCLGTGYLACARCSSTGSLVLVEPVATVNGADQPLSPPRTERCSNCSGAGKVMC     292
• CsOr-b      EKMRCKYCHGTGYLACARCSASGVFVSTEAVSIIGGCDRPLRPPSTQRCPNCSGAGKVMC     286
•             *: ***** ***********::* :* .* *: :.*.*:** ** *:** **********
•
• CsOr-a      PTCLCTGMAMASEHDPRIDPFD    314
• CsOr-b      PTCLCTGMVMASEHDPRINPFD    308
•             ********.*********:***
•
```

Supplemental Figure 1. Amino acid alignament of CsOr-a and CsOr-b.

Supplemental Figure 2

CsOr-a

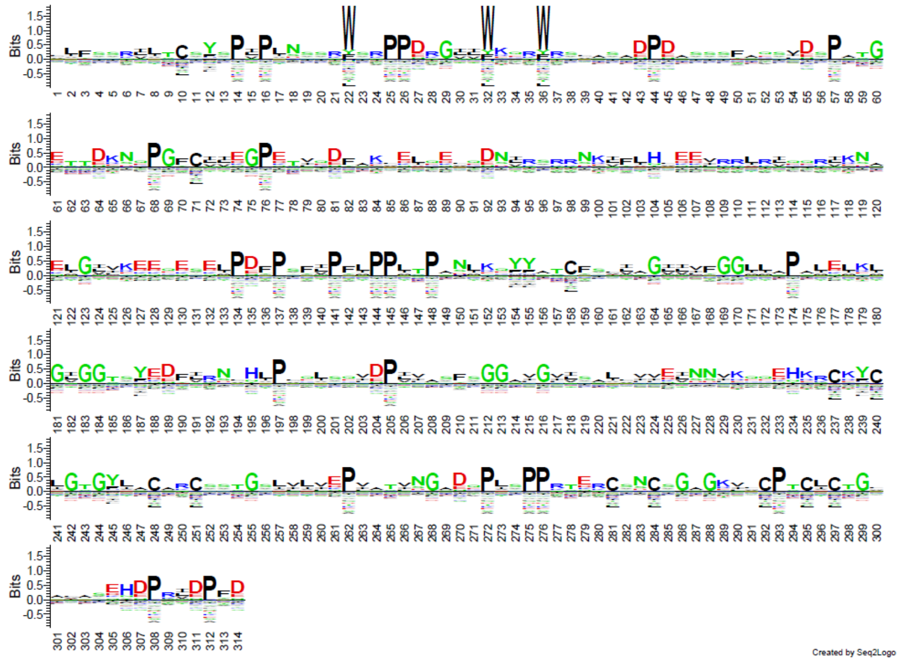

CsOr-b

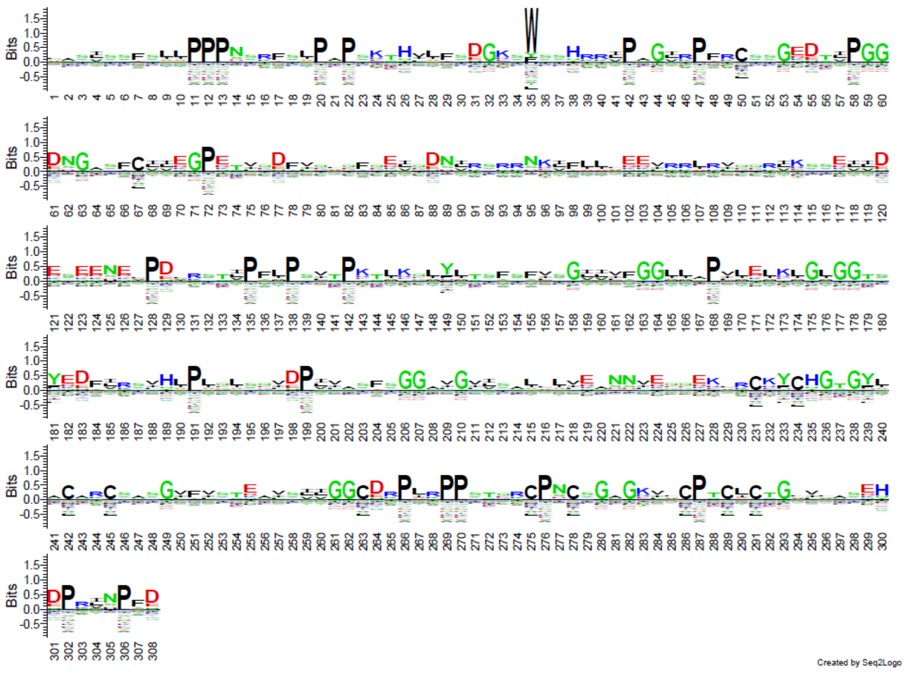

Supplemental figure 2. Consensus seqence of CsOr-a and CsOr-b obtained from blastP searches in the GenBank. The DnaJ-like cysteine-rich zinc finger domain that includes four repeats of the CxxCxGxG motif in the C-terminal region. Amino acids 281-288 in CsOr-a, and amino acids 276-282 in CsOr-b.

Supplemental Figure 3

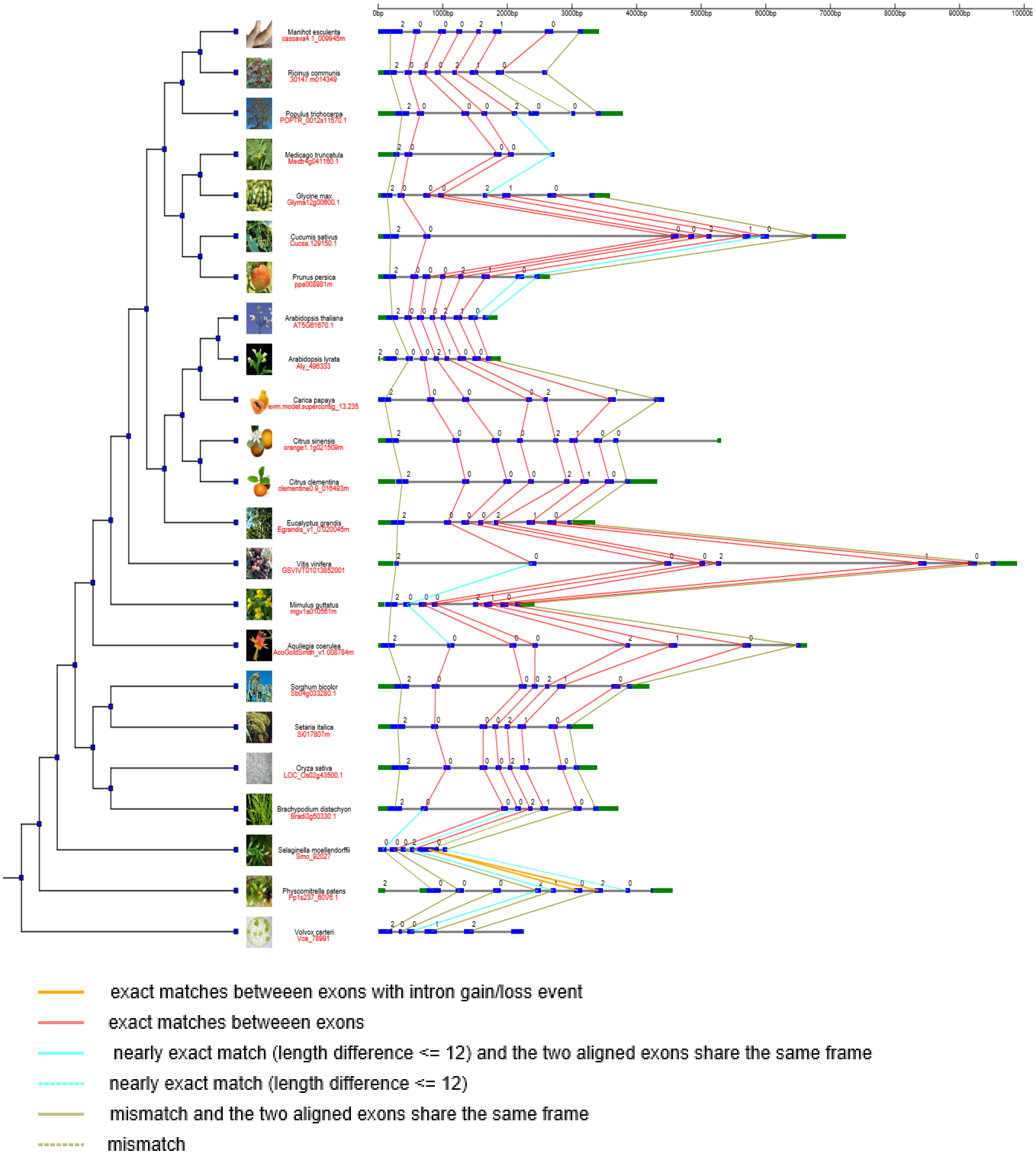

Supplemental Figure 3. Exalign was used for the orthologous gene structure evolution analysis of Or-a genes.

Supplemental Figure 4

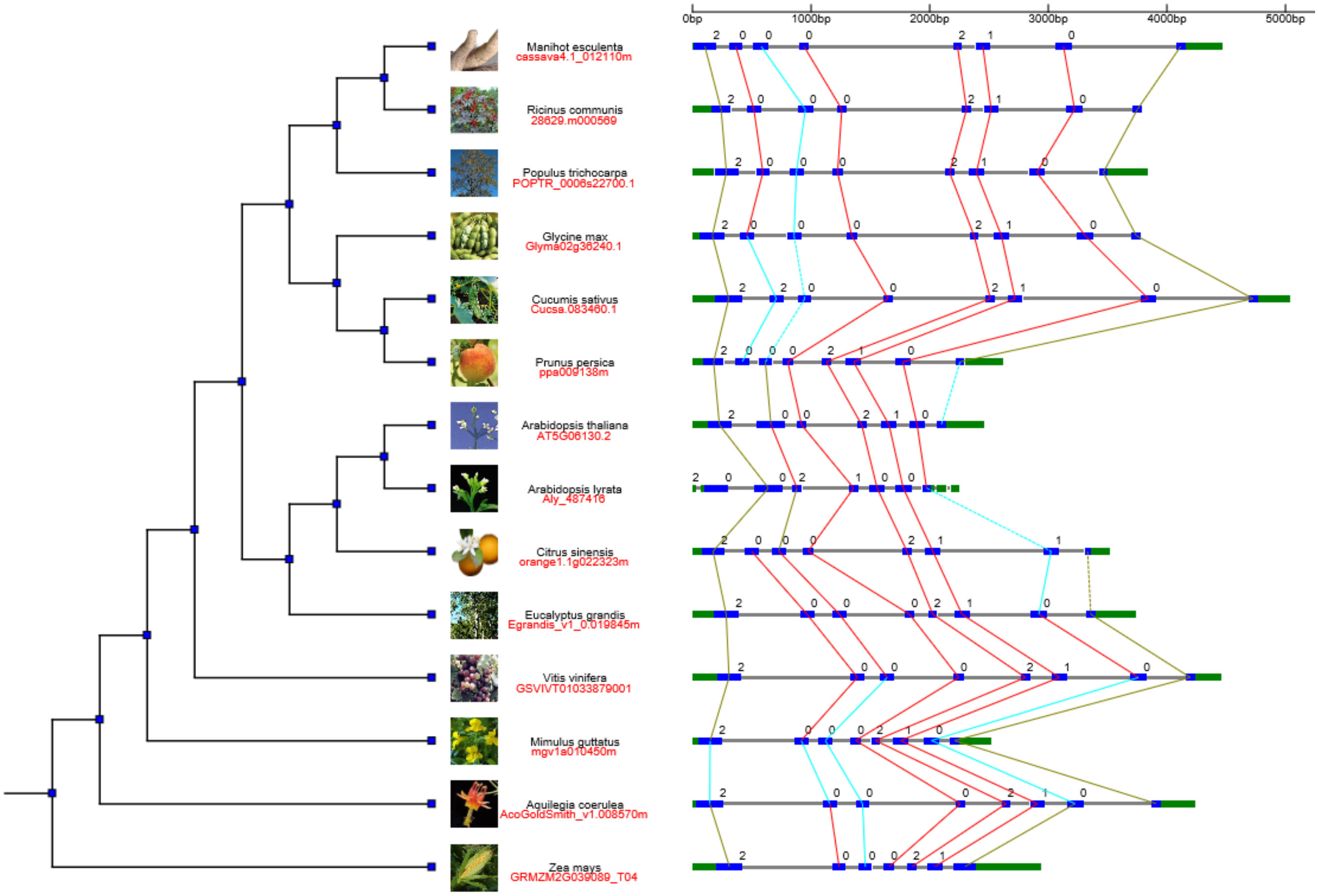

exact matches betweeen exons with intron gain/loss event

exact matches betweeen exons

nearly exact match (length difference <= 12) and the two aligned exons share the same frame

nearly exact match (length difference <= 12)

mismatch and the two aligned exons share the same frame

mismatch

Supplemental Figure 4. Exalign was used for the orthologous gene structure evolution analysis of Or-b genes.

Supplemental Figure 5

a

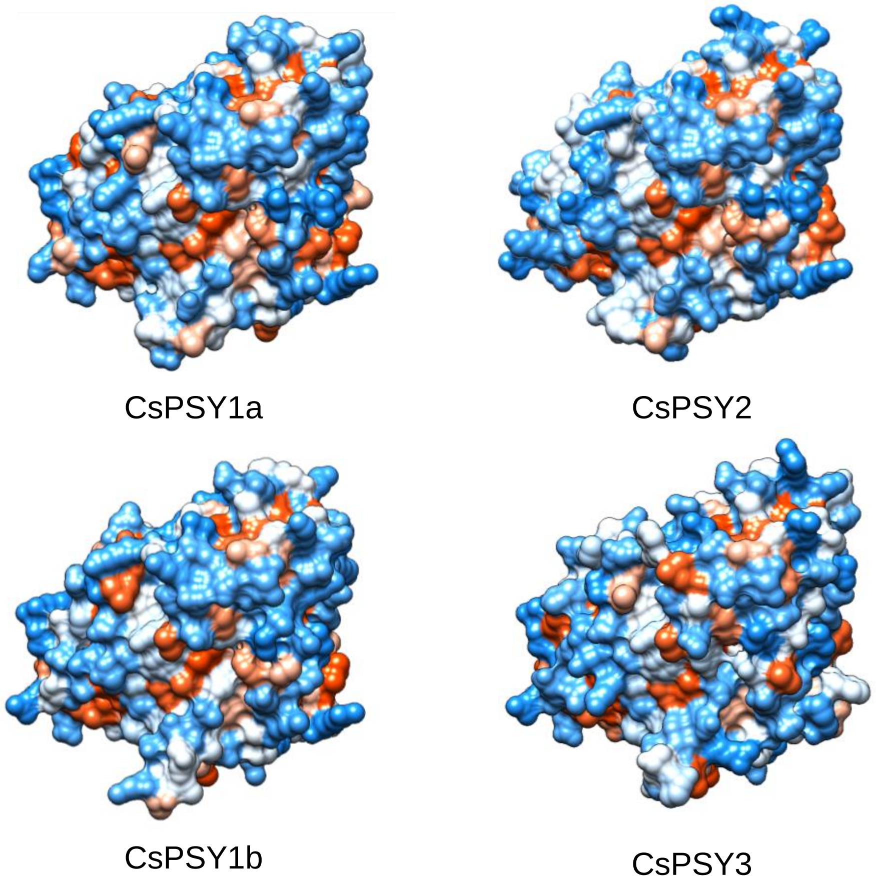

b

PSY protein	Hydrophobic residues %	Acidic residues %	Basic residues %	Neutral residues %	Instability index	Aliphatic index
CsPSY1a	48.57	13.14	13.71	24.58	51.88 (unstable)	88.37
CsPYS1b	48.70	13.11	13.39	24.80	50.92 (unstable)	89.52
CsPSY2	46.98	13.55	13.85	25.68	39.78 (stable)	88.19
CsPSY3	46.66	13.0	14.60	25.74	49.38 (unstable)	82.63

Supplemental Figure 5. Sequence and structural analyses of CsPSY proteins from saffron. (**a**) Tridimensional structures of CsPsy1a, CsPSY1b, CsPSY2 and CsPSY3. Blue colors correspond to the most hydrophilic, to white, to orange red for the most hydrophobic. Structures were generated using Chimera (https://www.cgl.ucsf.edu/chimera) (**b**) Stability, aliphatic index and percentage of hydrophobic, acidic, basic and neutral amino acids in the mature CsPSY proteins. The data were obtained with ProtParam (http://web.expasy.org).

Supplemental Figure 6

a

```
CsOr-a      MLFSSRILTCSYSPIPLN-----------------SSRWSRPPDRGIIWKQRWRSMASAD      43
AtOr-a      MSSLGRILSVSYPPDPYTWR---------------FSQYKLSSSLGRNRRLRWRFTAL-D      44
CsOr-b      MA---SISSFSL-LPPPNSRFSLPAPSKTHVLFSDGKSWSSH--RR--IP------AGIR      46
AtOr-b      ------MTCFSS-ATPHRHHLLLSSPSTSKSLLRFPSSYLKPSPSLLFHG------SSRS      47
                  :   *    *                    . :                 :

CsOr-a      PDASSSFAQSVDSPATGETTDKNQPGFCIIEGPETVQDFAKMELQEMQDNIRSRRNKIFL      103
AtOr-a      PESS--------SLDSESSADKFASGFCIIEGPETVQDFAKMQLQEIQDNIRSRRNKIFL      96
CsOr-b      PFRCSSGE-------DTIPGGDNGASFCIIEGPETVQDFVQMQFQEIQDNIRSRRNKIFL      99
AtOr-b      LLSCSDGSNNRPPPSDYLFGGYCFSNFCIIEGSETVQDFVQMQLQEIQDNIRSRRNKIFL      107
               .                .    .****** ******.:*::**:*************

CsOr-a      HMEEVRRLRIQQRIKNAELGIV-KEEQESELPDFPSFIPFLPPLTPANLKQYYATCFSMI      162
AtOr-a      HMEEVRRLRIQQRIKNTELGII-NEEQEHELPNFPSFIPFLPPLTAANLKVYYATCFSLI      155
CsOr-b      LMEEVRRLRVQQRIKSSEIIDES---EENEMPDMRSTIPFLPSVTPKTLKQLYLTSFSFV      156
AtOr-b      LMEEVRRLRVQQRIKSVKAINEDSELEATEMPEITSSIPFLPNVTPKTLKQLYSTSVALI      167
             ********:*****. :        :  *:*:: * ***** :*  .**  * *..:::

CsOr-a      AGIIVFGGLLAPALELKLGIGGTSYEDFIRNMHLPMQLSQVDPIVASFSGGAVGVISALM      222
AtOr-a      AGIILFGGLLAPTLELKLGIGGTSYADFIQSLHLPMQLSQVDPIVASFSGGAVGVISALM      215
CsOr-b      SGIIVFGGLLAPVLELKLGLGGTSYEDFIRSVHLPLQLSQVDPIVASFSGGAVGVISALM      216
AtOr-b      SGIIFFGGLIAPNLELKVGLGGTSYEDFIRSLHLPLQLSQVDPIVASFSGGAVGVISTLM      227
            :***.****:** ****:*:***** ***:.:***:*********************:**

CsOr-a      VVEINNVKQQEHKRCKYCLGTGYLACARCSSTGSLVLVEPVATVNGADQPLSPPRTERCS      282
AtOr-a      VVEVNNVKQQEHKRCKYCLGTGYLACARCSSTGALVLTEPVSAIAGGNHSLSPPKTERCS      275
CsOr-b      LVEANNVEQQEKMRCKYCHGTGYLACARCSASGVFVSTEAVSIIGGCDRPLRPPSTQRCP      276
AtOr-b      LIEVNNVKQQEKKRCKYCLGTGYLPCARCSASGVCLSIDPITRPRATNQLMQVATTKRCL      287
            ::* ***:***: ***** ***** *****::*  :  : ::   . :: :    *:**

CsOr-a      NCSGAGKVMCPTCLCTGMAMASEHDPRIDPFD      314
AtOr-a      NCSGAGKVMCPTCLCTGMAMASEHDPRIDPFD      307
CsOr-b      NCSGAGKVMCPTCLCTGMVMASEHDPRINPFD      308
AtOr-b      NCSGAGKVMCPTCLCTGMVTASEHDPRFDPFD      319
            ******************. *******::***
```

b

```
CsOr-a      ASADPDASSSFAQSVDSPATGETTDKNQPGFCIIEGPETVQDFAKMELQEMQDNIRSRRN      60
AtOr-a      -----------------------ADKFASGFCIIEGPETVQDFAKMQLQEIQDNIRSRRN      37
CsOr-b      -------CSSGED-------TIPGGDNGASFCIIEGPETVQDFVQMQFQEIQDNIRSRRN      46
AtOr-b      -------CSDGSNNRPPPSDYLFGGYCFSNFCIIEGSETVQDFVQMQLQEIQDNIRSRRN      53
                                    .    .****** ******.:*::**:*********

CsOr-a      KIFLHMEEVRRLRIQQRIKNAELGIV-KEEQESELPDFPSFIPFLPPLTPANLKQYYATC      119
AtOr-a      KIFLHMEEVRRLRIQQRIKNTELGII-NEEQEHELPNFPSFIPFLPPLTAANLKVYYATC      96
CsOr-b      KIFLLMEEVRRLRVQQRIKSSEIIDES---EENEMPDMRSTIPFLPSVTPKTLKQLYLTS      103
AtOr-b      KIFLLMEEVRRLRVQQRIKSVKAINEDSELEATEMPEITSSIPFLPNVTPKTLKQLYSTS      113
            **** ********:*****. :        :  *:*:: * ***** :*  .**  * *.

CsOr-a      FSMIAGIIVFGGLLAPALELKLGIGGTSYEDFIRNMHLPMQLSQVDPIVASFSGGAVGVI      179
AtOr-a      FSLIAGIILFGGLLAPTLELKLGIGGTSYADFIQSLHLPMQLSQVDPIVASFSGGAVGVI      156
CsOr-b      FSFVSGIIVFGGLLAPVLELKLGLGGTSYEDFIRSVHLPLQLSQVDPIVASFSGGAVGVI      163
AtOr-b      VALISGIIFFGGLIAPNLELKVGLGGTSYEDFIRSLHLPLQLSQVDPIVASFSGGAVGVI      173
            .::::***.****:** ****:*:***** ***:.:***:********************

CsOr-a      SALMVVEINNVKQQEHKRCKYCLGTGYLACARCSSTGSLVLVEPVATVNGADQPLSPPRT      239
AtOr-a      SALMVVEVNNVKQQEHKRCKYCLGTGYLACARCSSTGALVLTEPVSAIAGGNHSLSPPKT      216
CsOr-b      SALMLVEANNVEQQEKMRCKYCHGTGYLACARCSASGVFVSTEAVSIIGGCDRPLRPPST      223
AtOr-b      STLMLIEVNNVKQQEKKRCKYCLGTGYLPCARCSASGVCLSIDPITRPRATNQLMQVATT      233
            *:**::* ***:***: ***** ***** *****::*  :  : ::   . :: :    *

CsOr-a      ERCSNCSGAGKVMCPTCLCTGMAMASEHDPRIDPFD      275
AtOr-a      ERCSNCSGAGKVMCPTCLCTGMAMASEHDPRIDPFD      252
CsOr-b      QRCPNCSGAGKVMCPTCLCTGMVMASEHDPRINPFD      259
AtOr-b      KRCLNCSGAGKVMCPTCLCTGMVTASEHDPRFDPFD      269
            :** ******************. *******::***
```

Supplemental Figure 6. Multiple sequence alignment of Or proteins from saffron and Arabidopsis. (**a**) Including the predicted signal peptide. The transmembrane doamins are framed. (**b**) Without the predicted signal peptide.

Supplemental Figure 7

a

```
CsOr-a  115 QRIKNAELGIVKEEQEESELPDFPSFIPFL 144
AtOr-a  107 QRIKNTELGIINEEQEH-ELPNFPSFIPFL 135
CsOr-b  111 QRIKSSEII---DESEENEMPDMRSTIPFL 136
AtOr-b  119 QRIKSVKAINEDSELEATEMPEITSSIPFL 148
            ****   .         *   *:*  : * ****
```

b

Or-protein	Hydrophobic residues	Acidic residues	Basic residues	Neutral residues
CsOr-a	40.91	31.82	4.55	22.73
AtOr-a	36.98	26.32	5.26	31.58
CsOr-b	27.78	38.89	5.56	27.78
AtOr-b	42.11	31.58	5.26	21.05

c

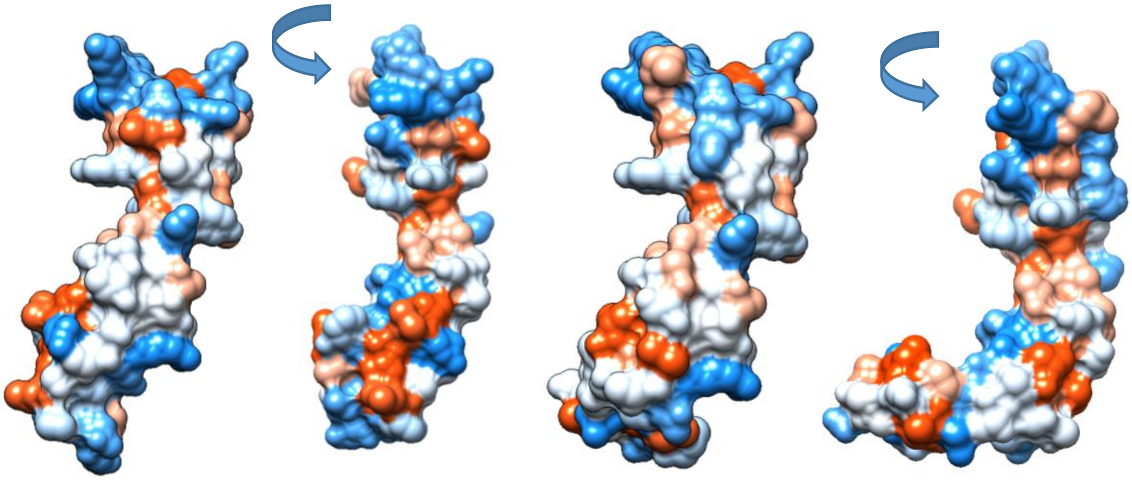

CsOr-a CsOr-b

Supplemental Figure 7. Sequence and structural analyses of Or proteins from saffron and Arabidopsis. (**a**) The proposed domain for interaction of Or with PSY proteins. (**b**) Percentage of hydrophobic, acidic, basic and neutral amino acids in the domain showed in A from the Or proteins from saffron and Arabidopsis. (**c**) Tridimensional structure of CsOr-a and CsOr-b generated with Chimera (https://www.cgl.ucsf.edu/chimera). Blue colors correspond to the most hydrophilic, to white, to orange red for the most hydrophobic.

Supplemental Table 1. Oligonucleotides used for isolation and studies on Or proteins from saffron.

	5′-3′	3′-5′
**cDNA amplification**		
**CsOr-a**	ATGGCTTCGGCCGATCCCGAT	TAATCAAATGGGTCGATC
**CsOr-b**	ATGTCCCTTCCCGCCCCTCCAA	TTAATCAAATGGGTTTATCCT
**Expression analyses**		
**CsOr-a**	ATGCTCTTTTCCTCTCGAATC	CATTAACTGTAGCGACAGGCT
**CsOr-b**	ATGGCTTCCATCTCCTCTTTC	GATTATAGAAACAGCTTCAGT
**CsPSY1a**	TTCGCCCATAGAATTTCCAA	TAAACCCTCTGCTCCGAAGA
**CsPSY1b**	GTTGTCTCACCGGTGGAAGT	TCACCAATTCGAGGTTGACA
**CsPSY2**	TTTCGGTCACCACCGATAAG	TCATGAGCAATGTTCCCAAG
**CsPSY3**	CACTATCCAGCTTCCCTCCA	CCACGACACCTCCTATAGCC
**Two hybrid assays**		
**CsOr-a**	gagtggccattatggcccATGGCTTCGGCCGATCCCGAT	ccgaggcggccgacatgGTAATCAAATGGGTCGATC
**CsOr-b**	gagtggccattatggcccATGTCCCTTCCCGCCCCTCCAA	ccgaggcggccgacatgTTAATCAAATGGGTTTATCCT
**CsPSY1a**	catggaggccgaattcATGGATGGGAACCTTGAGAGGAAG	gcaggtcgacggatccTCATCCTTTCAAGAGGCTCGA
**CsPSY1b**	catggaggccgaattcATGGTAGCGCCGAGTCTCGTAGCG	gcaggtcgacggatccCTAGGCATTACCTTGGCTACC
**CsPSY2**	catggaggccgaattcATGCCTTTAATTCCCTCAAAAGG	gcaggtcgacggatccTCAGACTTTTGAAGGACCAGC
**CsPSY3**	catggaggccgaattcATGAGTACCTCCATTTTACCAAATC	gcaggtcgacggatccCAAGTTAACAGCTTTAGCAAATCC
**CsCCD2**	catggaggccgaattcATGGCAAATAAGGAGGAGGCA	gcaggtcgacggatccTGTCTGCTTGGTGCTTCT
